# Non-Polio Enterovirus A71 and D68 Infection of Human Neuromuscular Organoids Reveals Distinct Mechanisms of Neuromuscular Impairment

**DOI:** 10.3390/v18080853

**Published:** 2026-08-04

**Authors:** Amber J. Schotting, Inés García-Rodríguez, Eline Freeze, Anoop T. Ambikan, Michael Wagner, Mira Mioch, Aymeric P. Y. L. Moffelein, William Jackson, Dasja Pajkrt, Katja C. Wolthers, Renata Vieira de Sá, Adithya Sridhar

**Affiliations:** 1 OrganoVIR Labs, Department of Paediatric Infectious Diseases, Emma Children’s Hospital, Amsterdam Institute for Reproduction and Development, Amsterdam Institute for Infection and Immunity, Amsterdam UMC, Location Academic Medical Center, University of Amsterdam, Meibergdreef 9, 1105 AZ Amsterdam, The Netherlands; 2 OrganoVIR Labs, Department of Medical Microbiology and Infection Prevention, Amsterdam Institute for Infection and Immunity, Amsterdam UMC, Location Academic Medical Center, University of Amsterdam, Meibergdreef 9, 1105 AZ Amsterdam, The Netherlands; 3 The Systems Virology Lab, Department of Laboratory Medicine, Division of Clinical Microbiology, Karolinska Institute, ANA Futura, Campus Flemingsberg, 17177 Stockholm, Sweden; 4 Department of Microbiology and Immunology and Center for Pathogen Research, University of Maryland School of Medicine, Baltimore, MD 21201, USA; 5 Emma Center for Personalized Medicine, Amsterdam UMC, Meibergdreef 9, 1105 AZ Amsterdam, The Netherlands

**Keywords:** non-polio enterovirus, A71, D68, acute flaccid myelitis, neuromuscular organoid, neuromuscular junction

## Abstract

Enterovirus A71 (EV-A71) and enterovirus D68 (EV-D68) are recognised as causative agents of severe neurological complications, including acute flaccid myelitis (AFM). However, the molecular mechanisms underlying the neurovirulence and effects on neuromuscular integrity remain poorly understood. Here, we employed human induced pluripotent stem cell-derived neuromuscular organoids (NMOs) to investigate the cellular tropism and pathogenic effects of EV-A71 and EV-D68 in a human-relevant context. Both viruses infected neuronal populations within NMOs, with EV-A71 exhibiting higher levels of viral replication than EV-D68. Transcriptomic analysis revealed downregulation of neuronal and muscular gene networks following infection. EV-A71 preferentially suppressed neuronal pathways, while both viruses exerted comparable effects on muscle-associated gene expression. These transcriptional changes highlighted alterations in pathways governing neuronal and muscle function and communication, prompting examination of synaptic vesicle machinery components. At the protein level, both viruses were associated with sporadic cleavage of the neuronal SNARE protein synaptosomal-associated protein 25 (SNAP25). In addition, infection with either virus increased cleaved caspase-3 levels, consistent with activation of apoptotic signalling. Together, these findings indicate virus-specific downstream effects and establish NMOs as a robust platform for dissecting enterovirus–host interactions relevant to AFM.

## 1. Introduction

Enteroviruses (EVs) represent one of the most prevalent groups of human pathogens worldwide and affect both paediatric and adult populations [1,2,3]. They cause a broad spectrum of clinical syndromes, including mild respiratory illness, hand, foot, and mouth disease (HFMD), and severe diseases such as myocarditis [1,2,4,5]. EVs are increasingly implicated in severe neurological complications, including encephalitis, meningitis, and acute flaccid myelitis (AFM), the latter resulting in muscle weakness and paralysis [6,7,8]. In recent years, non-polio EVs, notably EV-A71 and EV-D68, have emerged as important causes of paralytic viral neuropathies [9,10]. Despite their substantial disease burden, there are currently no approved antiviral therapies available against either virus, while a subgenogroup-specific vaccine (C4) against EV-A71 is used in China [11]. A deeper understanding of the pathogenic mechanisms underlying EV-induced neuromuscular injury is essential to support the development of targeted therapeutic strategies.

Clinically, EV-A71 and EV-D68 are associated with overlapping yet distinct disease presentations. EV-A71 is more frequently linked to aseptic meningitis and brainstem encephalitis [12,13,14], whereas EV-D68 is strongly associated with outbreaks of AFM, usually following a respiratory illness [14,15]. Although both viruses are implicated in paralytic disease in humans [14], whether they differ in their underlying mechanisms of neuromuscular injury remains unclear. Human post-mortem studies and infection studies in mouse models suggest that EV-A71 is strongly neurotropic, with infection of motor neurons (MNs) in the spinal cord and brainstem representing a major determinant of central nervous system (CNS) pathology [16,17]. Additionally, these studies indicate that EV-A71 can access the CNS via retrograde transport, consistent with MN disease [18,19,20,21]. In contrast, EV-D68-associated disease in humans is characterised by pathology in the anterior horn cells of the spinal cord and loss of MNs [15]. Consistent with this clinical presentation, studies in cell cultures and mice indicate that EV-D68 can infect MNs and induce paralytic phenotypes, as viral replication was detected in both the spinal cord and peripheral neuromuscular tissues [22,23]. Notably, only EV-D68, and not EV-A71, infection has been reported to cause pronounced skeletal muscle pathology in mice, where muscle-restricted infection alone can be sufficient to drive paralysis even in the absence of detectable spinal cord involvement [24]. More recently, human iPSC-derived skeletal muscle systems have demonstrated robust EV-D68 myotropism and associated muscle fibre damage [25].

These findings highlight shared and divergent patterns of neuromuscular tropism while raising questions regarding virus-specific mechanisms of paralysis in humans. Human stem cell-derived neural, muscular, and multilineage (neuromuscular) organoids provide a powerful platform to address these questions by enabling the study of EV-A71 and EV-D68 infection in a human, tissue-relevant context. Such models allow direct interrogation of viral tropism and host responses in MNs and have already proven effective for studying neurotropic RNA viruses, including parechovirus-3, where virus-specific immune, metabolic, and excitotoxic pathways were identified [26,27].

Here, we employed three-dimensional (3D) human multilineage neuromuscular organoids (NMOs) that include the neuronal and muscle components and recapitulate key aspects of human neuromuscular junction (NMJ) associated function [28]. Using this platform, we investigated host transcriptional responses to EV-A71 and EV-D68 infection across neuronal and muscular compartments. Transcriptomic analyses identified infection-induced alterations not only in pathways governing neuronal and muscle function but also in communication, providing a rationale to examine components of the synaptic vesicle machinery. We, therefore, focused also on the neuronal SNARE protein synaptosomal-associated protein 25 (SNAP25), which is essential for synaptic transmission at the NMJ [29,30,31]. To assess potential consequences of viral infection on neuronal integrity, we examined the apoptotic marker cleaved caspase-3 (CC3). This study leverages human NMOs as a physiologically relevant system to dissect EV–host interactions in neuromuscular tissue.

## 2. Materials and Methods

### 2.1. Cell Models

#### 2.1.1. Human Induced Pluripotent Stem Cell (hiPSC) Culture

Three hiPSC lines, IMR90-4, (WiCell, WISCi004-B (RRID: CVCL_C437)), M001 (STEMCELL™ Technologies, STiPS-M001, (RRID:CVCL_9S50)) and Gibco^®^ A18945 (ThermoFisher Scientific, (RRID: CVCL_RM92)), were cultured on 6-well plates coated with GelTrexTM (Cat. #A1413301; ThermoFisher Scientific, Waltham, MA, USA) or LN521 (Cat. # LN321-05; BioLamina, Sundbyberg, Sweden). All lines were maintained in mTeSR™ Plus (Cat. #100-0276; STEMCELL Technologies, Vancouver, BC, Canada) supplemented with 1% (*v*/*v*) Pen-Strep at 37 °C, 5% CO_2_, and 95% humidity with daily medium changes. hiPSCs were passaged weekly with ReLeSRTM (Cat. #100-0483; STEMCELL™ Technologies, Vancouver, BC, Canada), and hiPSC lines were routinely tested for mycoplasma and confirmed to be negative using the MycoAlert^®^ Mycoplasma Detection Kit (Cat. #LT07-318; Lonza, Basel, Switzerland).

#### 2.1.2. hiPSC Motor Neuron Culture

hiPSC-MNs were generated with the STEMdiff™ MN Differentiation Kit (Cat. #100-0871; STEMCELL™ Technologies, Vancouver, BC, Canada) and STEMdiff™ MN Maturation Kit (Cat. #100-0872; STEMCELL™ Technologies, Vancouver, BC, Canada) following the manufacturer’s instructions. Briefly, IMR90-4 hiPSCs were dissociated into a single cell suspension using ACCUTASE™ (Cat. #07922; STEMCELL™ Technologies, Vancouver, BC, Canada). The cells were resuspended in MN Differentiation Medium 1 (STEMCELL Technologies) supplemented with 10 µM Y-27632 (Cat. #10005583; Cayman Chemicals, Ann Arbor, MI, USA). hiPSCs were seeded at a density of 360,000 cells/well (300 cells/microwell) in a 24-well AggreWell™ 400 plate (Cat. #34411; STEMCELL™ Technologies, Vancouver, BC, Canada), which had been pre-treated with anti-adherence rinsing solution (Cat. #07010; STEMCELL™ Technologies, Vancouver, BC, Canada). The cells were incubated at 37 °C with 5% CO_2_ and 95% humidity for 2 days to promote the formation of embryoid bodies (EBs). On day 2, 75% of the medium was replaced daily with MN Differentiation Medium 2 (STEMCELL Technologies). From day 4 to day 9, medium was replaced daily with MN Differentiation Medium 3 (STEMCELL Technologies). On day 9, the EBs, consisting of neural progenitors, were harvested, and dissociated using ACCUTASE™. The neural progenitors were seeded onto 24-well plates pre-coated with 15 µg/mL Poly-L-Ornithine (Cat. #P4957-50ML; Sigma-Aldrich, Saint-Louis, MO, USA) and 10 µg/mL laminin (Cat. #L2020, Sigma-Aldrich, Saint-Louis, MO, USA). Cells were plated at a density of 50,000 cells/well in MN Differentiation Medium 4 (STEMCELL Technologies) supplemented with 10 µM Y-27632. On day 11, 75% of the medium was replaced with MN Differentiation Medium 4. Starting from day 14, hiPSC-MNs were cultured in MN Maturation Medium (STEMCELL Technologies) until day 28, with 75% medium changes occurring every 2–3 days. hiPSC-MNs were consistently maintained at 37 °C and 5% CO_2_ and were considered mature by day 28.

#### 2.1.3. Generation of Neuromuscular Organoids

NMOs were generated from hiPSCs following the protocol by Faustino Martins et al. [28]. Briefly, hiPSCs were dissociated into single cells using ACCUTASE™ and seeded onto GelTrex™-coated 6-well plates in neurobasal medium supplemented with 10 μM Y-27632, 3 μM CHIR99021 (Cat. #4423; R&D Systems, Minneapolis, MN, USA), and 40 ng/mL bFGF (Cat. # 100-18B; PeproTech, Cranbury, NJ, USA). Seeding densities were optimized for each line: IMR90-4 and Gibco^®^ A18945 at 125,000 cells/cm^2^, and M001 at 75,000 cells/cm^2^. After three days of daily medium changes, neuromesodermal progenitors (NMPs) were dissociated and seeded (4500–9000 cells/well) into ultra-low binding 96-well plates with NB medium containing Y-50 μM Y-27632, 10 ng/mL bFGF, 2 ng/mL IGF1 (Cat. #100-11; PeproTech, Cranbury, NJ, USA), and 2 ng/mL HGF (Cat. #315-23 PeproTech, Cranbury, NJ, USA). Plates were centrifuged to promote aggregation. Two days later, half the medium was replaced with fresh NB medium containing IGF1 and HGF. From day 4, NMOs were maintained in NB medium without additional growth factors, with medium changes every other day. On day 10, NMOs were transferred to 60 mm dishes, and on day 30 to 100 mm dishes, both pre-coated with anti-adherence solution. NMOs were cultured on an orbital shaker at 75 rpm, 37 °C, 5% CO_2_, and 95% humidity, with medium changes every 2–3 days. NMOs were considered mature by day 50.

### 2.2. Virus Strains and Infection

#### 2.2.1. Virus Strains and Propagation

EV-A71 C2 (accession #FJ172159) was a gift from Dr Sylvie Alonso and was isolated from a clinical specimen at the National University of Singapore [32]. EV-D68 Fermon (accession #AY426531) was obtained from Dr Adam Meijer from the National Institute for Public Health and the Environment (RIVM, Utrecht, The Netherlands). The EV-D68 Fermon strain was selected based on preliminary screening in human motor neuron cultures, where it showed the most consistent replication compared to other EV-D68 strains tested. Both strains were propagated in RD cells. The 50% tissue culture infectious dose (TCID_50_) of the virus stocks was calculated according to the Reed and Muench method [33]. The virus stocks were aliquoted and stored at −70 °C.

#### 2.2.2. Viral Infections

##### hiPSC-MNs Viral Infection

Mature hiPSC-MNs were inoculated with 10^5^ TCID_50_ of the viruses in 200 µL of MN Maturation Medium. The viral inoculation was incubated for 2 h at 37 °C with 5% CO_2_ and 95% humidity. Subsequently, the viral inoculum was removed, and the hiPSC-MNs were washed three times with DMEM/F12 medium (Gibco, Waltham, MA, USA). Medium was replenished, and hiPSC-MNs were incubated for 10 min, after which the 0 h post infection (hpi) time point was collected. Medium and protein collection was performed at different time points, and fresh media were replenished after each collection.

##### NMOs Viral Infection

hiPSC-NMOs were transferred to a round-bottom 96-well plate, previously coated with anti-adherence rinsing solution, and inoculated with 10^5^ TCID_50_ in 100 µL per virus. The NMOs were incubated for 2 h at 37 °C with 5% CO_2_. Thereafter, the inoculum was removed, and the NMOs were washed three times with DMEM/F12. The NMOs were moved to 48-well plates, previously coated with anti-adherence rinsing solution, with 500 µL NB medium in each well. The plates were incubated for 10 min at 75 rpm at 37 °C with 5% CO_2_, after which the 0 hpi time point was collected. Medium was collected at 24, 48, and 72 hpi, with fresh media replenished after each time point, while protein samples were collected at 96 and 168 hpi.

#### 2.2.3. BoNT/A Treatment

For the BoNT/A (Xeomin; Merz Pharmaceuticals GmbH, Frankfurt, Germany) condition, 4 Units of BoNT/A were added to the wells every 24 h, and washing was omitted until protein collection.

### 2.3. Immunofluorescence Staining

hiPSC-MNs were fixed using 4% (*v*/*v*) formaldehyde (FA; Cat. #252549; Sigma-Aldrich, Saint Louis, MO, USA) for 10 min at room temperature (RT) and were used directly for immunostaining. NMOs were fixed in 4% (*v*/*v*) FA for 30 min at RT.

NMOs were washed three times with PBS and incubated in 30% (*w*/*v*) sucrose (Cat. #1.07687.0250; Merck–Millipore corporation, Burlington, VT, USA) at 4 °C overnight for cryoprotection. NMOs were embedded in Tissue-Tek^®^ O.C.T. (Cat. #4583; Sakura Finetek, Tokyo, Japan), snap frozen on dry ice, and stored at −70 °C. Frozen NMOs were cryosectioned at 20 μm thickness using a Cryostat NX70 (ThermoFisher Scientific, Waltham, MA, USA) on SuperFrost™ Plus Adhesion Microscope Slides (Cat. #J1800AMNZ; Epredia, Portsmouth, UK) and stored at −70 °C until staining. Prior to staining, NMO sections were air-dried at RT and rehydrated with PBS.

For both hiPSC-MN and NMOs blocking was performed using blocking buffer containing 1% (*v*/*v*) Triton X-100 (Cat. #X100-500ML; Sigma-Aldrich, Saint-Louis, MO, USA) and 10% Fish Serum Blocking Buffer (Cat. #37527; ThermoFisher Scientific, Waltham, MA, USA) in PBS for 1 h at RT. Slides were incubated with primary antibodies (Table 1) in a 1:1 dilution of the blocking buffer in PBS and incubated overnight at 4 °C in a humidified chamber. The following day, slides were washed three times with PBS for 5 min and incubated for 2 h at RT with the secondary antibodies (Table 1) and Hoechst (Cat. #H3570; Invitrogen, Waltham, MA, USA) for nuclear staining. After three additional washes in PBS, sections were mounted using ProLong™ Glass Antifade Mountant (Cat. #P36984, ThermoFisher Scientific, Waltham, MA, USA). Slides were imaged using a Leica TCS SP8-X microscope and Leica LAS AF V4.0 Software (Leica Microsystems). Image processing and analysis were performed using LAS-X 3D software (https://www.leica-microsystems.com/products/microscope-software/p/leica-las-af-3d-analysis/ accessed on 28 July 2025) (Leica Microsystems, Wetzlar, Germany).

### 2.4. Western Blot

NMOs were washed once with PBS and lysed using NP-40 lysis buffer (Cat. #J60766.AK; ThermoFisher Scientific, Waltham, MA, USA) supplemented with cOmplete™ Protease Inhibitor Cocktail (1:25) (Cat. #11697498001; Roche, Basel, Switzerland). The NMOs were mechanically lysed by pipetting and incubated on ice for 10 min to facilitate lysis. Lysates were centrifuged at 300 *g* for 20 min at 4 °C, and the resulting supernatant was stored at −70 °C. Total protein concentration was determined using the PierceTM BCA Protein Assay kit (Cat. #23227; ThermoFisher Scientific, Waltham, MA, USA) following the manufacturer’s instructions.

Samples were mixed with 2× Laemmli Sample Buffer (Cat. #1610737; Bio-Rad Laboratories Inc., Hercules, CA, USA) containing 5% (*v*/*v*) β-mercaptoethanol (Cat. #1610710; Bio-Rad Laboratories Inc., Hercules, CA, USA) and denatured at 99 °C for 10 min. Proteins were separated by SDS-PAGE using 10% Mini-PROTEAN^®^ precast gels (Cat. #4568033; Bio-Rad Laboratories Inc., Hercules, CA, USA) in Tris/Glycine/SDS buffer (Cat. #1610732; Bio-Rad Laboratories Inc., Hercules, CA, USA) at 4 °C. Subsequently, proteins were transferred at RT onto nitrocellulose membranes (Cat. #1620145; Bio-Rad Laboratories Inc., Hercules, CA, USA) in Tris/Glycine buffer (Cat. #1610734; Bio-Rad Laboratories Inc., Hercules, CA, USA) containing 20% (*v*/*v*) methanol (Cat. #ZY10016; MLS, Menen, Belgium). Membranes were blocked with 5% (*w*/*v*) non-fat skimmed (Cat. #A0830.0500; ITW Reagents, Castellar del Vallès, Spain) in PBS containing 0.1% (*v*/*v*) Tween-20 (PBS-T; Cat. #P1379; Sigma-Aldrich, Saint-Louis, MO, USA) for 30 min at RT. After blocking, membranes were incubated overnight at 4 °C with primary antibodies (Table 2) in 2.5% (*w*/*v*) non-fat powder milk in PBS-T. Following three washes with PBS-T, membranes were incubated with horseradish peroxidase (HRP)-conjugated IgG secondary antibodies for 2 h at RT. Thereafter, the membranes were washed three times with PBS-T. Protein bands were visualized using Clarity™ Western ECL Substrate (Cat. #1705060; Bio-Rad Laboratories Inc., Hercules, CA, USA) according to the manufacturer’s instructions. Imaging was performed using the ImageQuantTM LAS 4000 (GE Healthcare, Chicago, IL, USA).

Band intensities were quantified using ImageJ software version 1.54g (National Institutes of Health, Bethesda, MD, USA). Relative protein expression was calculated by normalizing the intensity of each target band to the corresponding loading control.

Statistical analysis was performed using GraphPad Prism 8 (GraphPad Software Inc., San Diego, CA, USA). Data represent the geometric mean± geometric standard deviation (SD). Statistical significance of the protein abundance was assessed using Kruskal–Wallis test with Dunn’s multiple comparisons. A *p*-value < 0.05 was considered statistically significant and is marked with an asterisk in the graphs. *p*-values below 0.01, 0.001, and 0.0001 are indicated by two, three, and four asterisks, respectively.

### 2.5. Quantitative RT-qPCR

Total RNA was isolated using the PureLinkTM RNA Mini kit (Cat. #12183025; Invitrogen, Waltham, MA, USA) according to the manufacturer’s instructions. From the eluted RNA, cDNA synthesis was performed by adding 10 μL of reverse transcriptase mastermix consisting of 10× CMB1-buffer (pH 8.3), 100 mM MgCl_2_, 20 mg/mL α-casein, 40 mM dNTPs (10 mM each; Roche, Basal, Switzerland), 1.5 μg/μL Primer Random Hexamers (Cat. #11034731001; Roche, Basal, Switzerland), 40 U/μL Recombinant RNasin^®^ Ribonuclease Inhibitor (Cat. #N2111; Promega, Madison, WI, USA), and 200 U/μL SuperScript II (Cat. #18064014; Invitrogen, Waltham, MA, USA). Samples were incubated at 42 °C, 350 rpm for 30 min. cDNA was stored at −20 °C.

RT-qPCR was performed using a LightCycler^®^ 480 SYBR Green I Master (Cat. #04707516001; Roche, Basel, Switzerland) for EV-A71 and a TaqMan (LightCycler^®^ 480 Probes Master, Cat. #04887301001; Roche, Basel, Switzerland) for EV-D68 samples. All samples were run on a Bio-Rad CFX384™ Connect Real-Time PCR Detection System as previously described [34]. Cq values were transformed into viral genome copies using a standard curve for each specific set of primers. The primers used in this study are listed in Table 3.

Statistical analyses were performed using GraphPad Prism 8 (GraphPad Software Inc.). Data are presented as geometric mean ± geometric standard deviation (GSD), unless stated otherwise. Statistical significance for relative increases in viral RNA copy numbers was assessed using a Kruskal–Wallis test followed by Dunn’s multiple comparisons test, while differences in viral titres were analysed using a Wilcoxon test. A *p* value < 0.05 was considered statistically significant. Significance levels are indicated in the figures as follows: *p* < 0.05 (*), *p* < 0.01 (**), *p* < 0.001 (***), and *p* < 0.0001 (****).

### 2.6. RNA-Sequencing and Transcriptome Analysis

#### 2.6.1. RNA Sequencing

RNA sequencing was performed by Macrogen Europe BV (Amsterdam, The Netherlands). RNA integrity and concentration were assessed using an Agilent 2100 Bioanalyzer (Agilent Technologies, Santa Clara, CA, USA). Libraries were prepared using the Watchmaker mRNA Library Prep kit (Illumina, San Diego, CA, USA) by the manufacturer. Library quality and fragment size distribution were assessed using a Fragment Analyzer system, according to the service provider’s standard workflow. Clustering, sequencing, and primary data processing were performed by the sequencing provider on the NovaSeq X platform (Illumina), generating paired-end reads.

Raw sequencing reads were first subjected to quality control with FastQC v0.11.9. Adapter sequences and low-quality bases were removed using Trimmomatic v0.38. Cleaned reads were aligned to the human reference genome (GRCh38) using HISAT2 v2.1.0 with default parameters. Transcript assembly and quantification were performed with StringTie v2.1.3b to obtain gene-level counts, FPKM, and TPM values.

#### 2.6.2. Data Analysis

The differential expression was determined with DESeq2 v.1.50.2 using the raw count files. Differential expressed genes with a *p*-adjusted value of <0.05 were considered significant. The PCA plots were generated using the web-based platform NASQAR DEBrowser [35]. The volcano plots and heatmap were generated with R v.4.5.1 packages ggplot2 v4.0.0. The IDs of all genes were selected from the significant gene lists and Venn diagrams were created using a web tool (https://bioinformatics.psb.ugent.be/webtools/Venn/ accessed on 28 July 2025) from the University of Gent.

#### 2.6.3. GSEA

Gene set enrichment analysis (GSEA) was performed using the clusterProfiler R package (v4.10.0). Genes were ranked by their DESeq2-derived Wald statistics, and enrichment was assessed against the Gene Ontology Biological Process (GO BP) database using the GSEA algorithm with parameters minGSSize = 3, maxGSSize = 800, and pAdjustMethod = “BH” [36,37].

To focus on pathways relevant to neuromuscular biology, we used a custom subset of GO BP terms containing processes related to neuronal and muscle function (e.g., “synaptic signalling”, “muscle contraction”, “axon guidance”). These terms were extracted from the Human MSigDB GO database v2025.1.Hs (June 2025) and formatted into a .gmt file for use as the reference gene set collection. Dot plots and text files of enriched GO-terms were exported.

## 3. Results

### 3.1. NMOs Recapitulate Neuromuscular Architecture and Support Infection of EV-A71 and EV-D68

**Figure 1:**
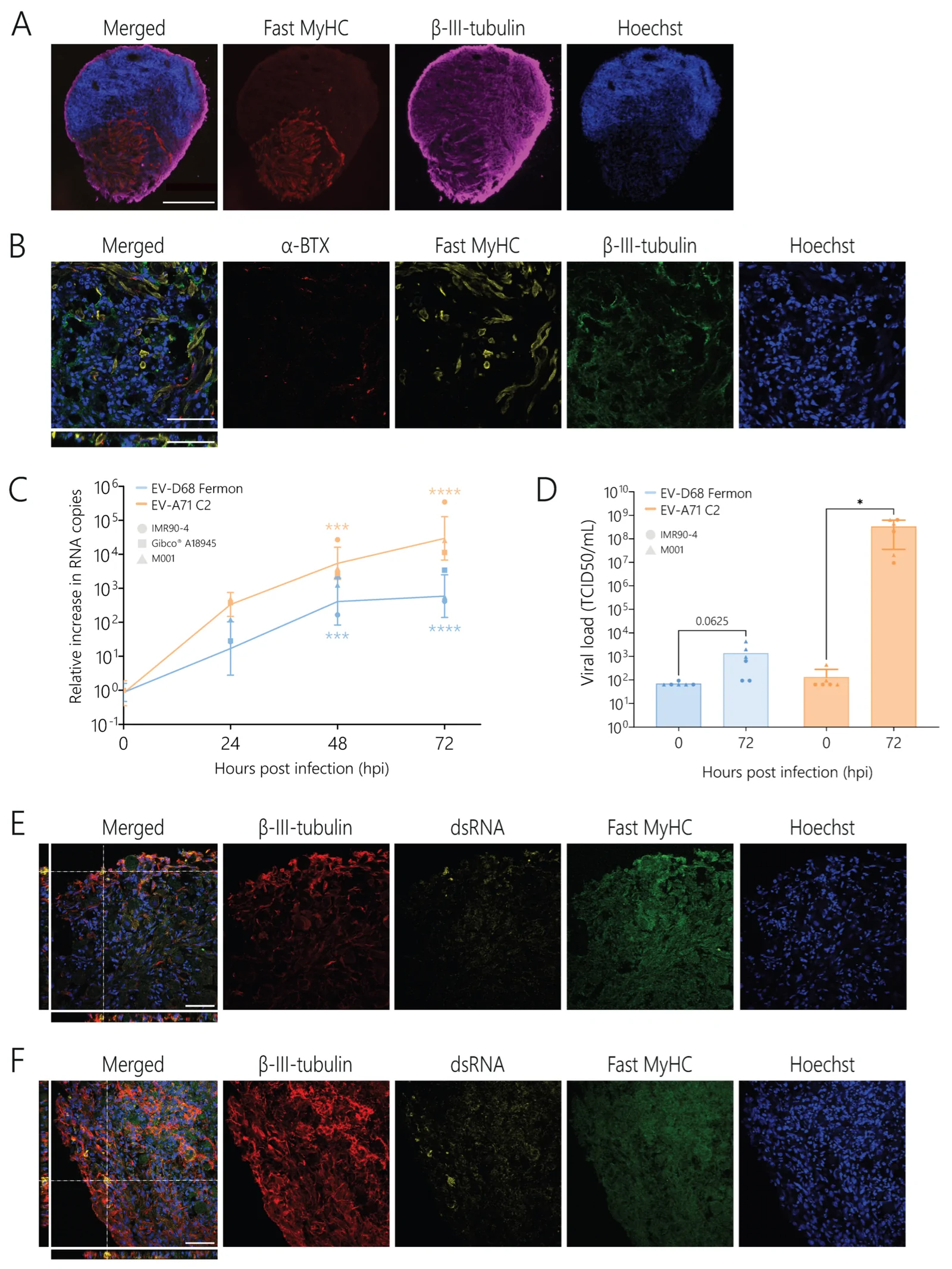
NMOs recapitulate neuromuscular architecture and support EV infection. (**A**) Representative immunofluorescence image of an IMR90-4-derived NMO stained for Hoechst, β-III-tubulin (TUJ1, neurons) and fast myosin heavy chain (Fast MyHC; muscle). Scale bars: 300 µm. (**B**) Confocal z-stack of a NMJ in an IMR90-4-derived NMO stained with Hoechst, α-bungarotoxin (α-BTX; nAChRs), β-III-tubulin (TUJ1), and Fast MyHC. The main panel shows the XY projection, with the orthogonal YZ view displayed on the bottom edge. Scale bars: 50 µm and 20 µm. (**C**) Relative increase in viral RNA copy numbers in the supernatant at indicated time points following infection with EV-A71 (0 vs. 24 hpi: *p* = 0.21; 0 vs. 48 hpi: *** *p* = 0.0003; 0 vs. 72 hpi: **** *p* < 0.0001) or EV-D68 (0 vs. 24 hpi: *p* = 0.16; 0 vs. 48 hpi: *** *p* = 0.0001; 0 vs. 72 hpi: **** *p* < 0.0001). Data represent the geometric mean of three technical replicates per condition, calculated separately for each hiPSC line: IMR90-4 (circles), Gibco^®^ A18945 (squares), and M001 (triangles). Statistical significance was assessed per virus using a Kruskal–Wallis test with Dunn’s multiple comparisons test. (**D**) Viral titres of EV-A71 (* *p* < 0.05) and EV-D68 (*p* = 0.06) in the supernatant at 0 and 72 hpi. Data represent the geometric mean of three technical replicates per condition, calculated separately for each hiPSC line (IMR90-4 and M001). Statistical significance was assessed per virus using a Wilcoxon test. (**E**,**F**) Confocal z-stacks of Gibco^®^ A18945-derived NMOs at 72 hpi infected with EV-D68 (**E**) or EV-A71 (**F**), stained for Hoechst, Fast MyHC, dsRNA, and β-III-tubulin (TUJ1). The main panels show XY projections, with orthogonal XZ and YZ views displayed on the left and bottom edges, respectively, to visualize the three-dimensional distribution of the signal. Scale bars: 50 µm.

NMOs were generated from three different human induced pluripotent stem cell (hiPSC) lines (IMR90-4, M001, and Gibco^®^ A18945). Characterisation to confirm their cellular composition and organisation was performed using immunofluorescence staining. Expression of the neuron-specific microtubule protein β-III-tubulin (TUJ1) confirmed the establishment of a neuronal compartment [38,39], while a spatially distinct region expressing fast myosin heavy chain (Fast MyHC) indicated the presence of a mature muscular compartment [40,41,42]. Notably, neurons innervated the muscle region (Figure 1A). These findings were consistent across all NMOs, irrespective of the hiPSC line (Supplementary Figure S1A). To assess the formation of NMJs, NMOs were stained with α-bungarotoxin (α-BTX), which selectively binds nicotinic acetylcholine receptors (nAChRs) [43,44]. Immunofluorescence analysis revealed α-BTX co-localisation with neurons, suggesting the establishment of NMJs within the organoid (Figure 1B). Contractions were observed in some NMOs, indicating the presence of a functional muscle compartment; however, this phenotype was not consistent across all hiPSC lines and batches (Video S1).

We evaluated the susceptibility of NMOs to EV infection, their capacity to support viral replication, and viral cellular tropism. NMOs were infected with 10^5^ TCID_50_ of EV-A71 or EV-D68. RT-qPCR analysis revealed a significant increase in viral genome copies over time for both EV-A71 (~10^4^ at 72 h post infection (hpi)) and EV-D68 (~10^2^ at 72 hpi), with a ~100-fold greater increase observed in EV-A71-infected NMOs compared to EV-D68-infected NMOs (Figure 1C). The number of infectious viral particles increased over time for both viruses. EV-A71 reached markedly higher final titres (10^8^ TCID_50_) compared to EV-D68 (10^4^ TCID_50_) in NMOs after 72 hpi (Figure 1D). Immunofluorescent staining performed at 72 hpi revealed that dsRNA, a marker of active viral RNA replication, was predominantly localised within neuronal cells in both EV-A71- and EV-D68-infected NMOs (Gibco^®^ A18945; Figure 1E,F), whereas it was absent in mock conditions (Supplementary Figure S1B).

These results demonstrate that NMOs develop distinct neuronal and muscular compartments, establish functional junctions, and are permissive to infection by both EV-A71 and EV-D68.

### 3.2. EV-A71 and EV-D68 Differentially Downregulate Neuronal and Muscular Pathways

**Figure 2:**
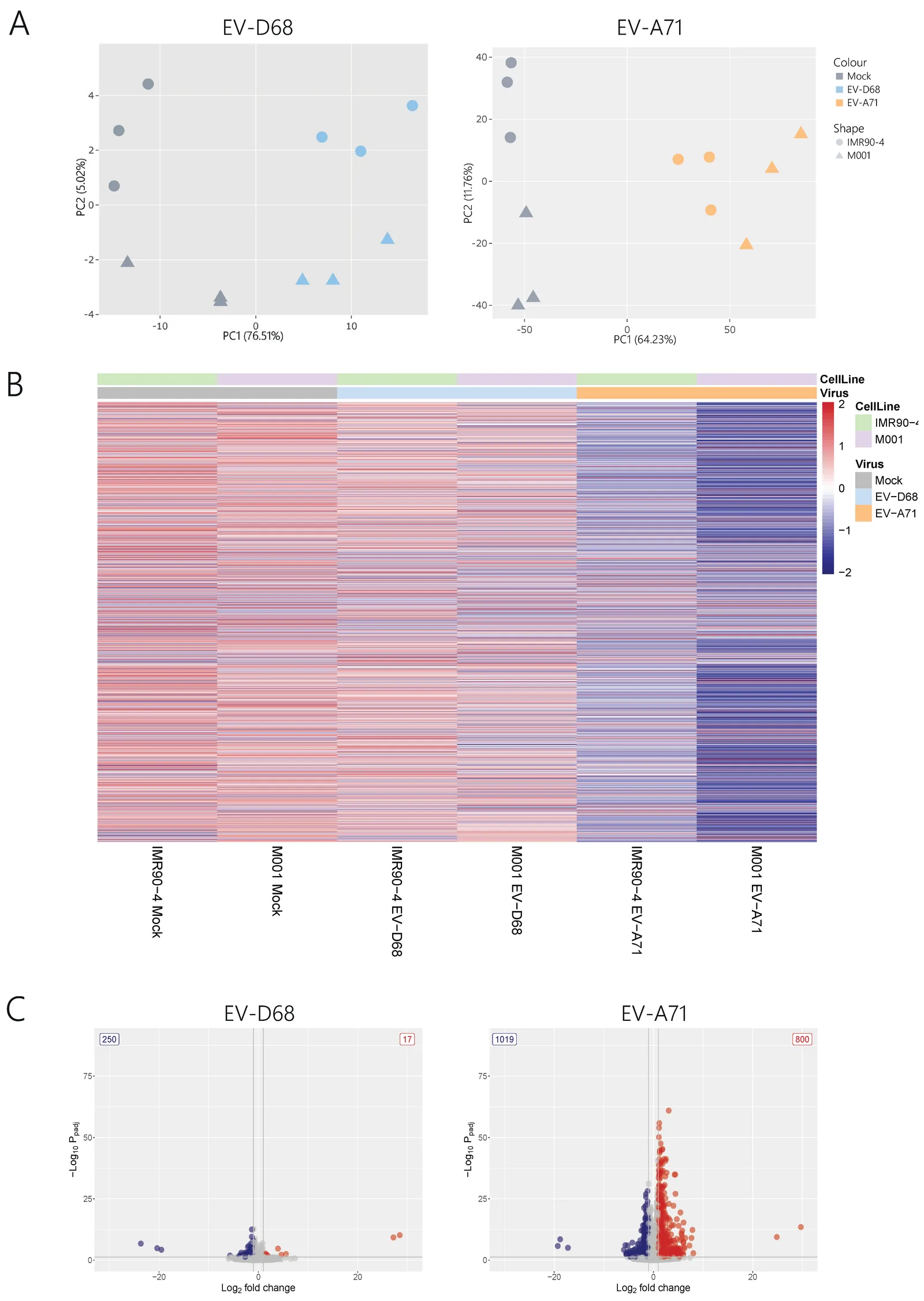

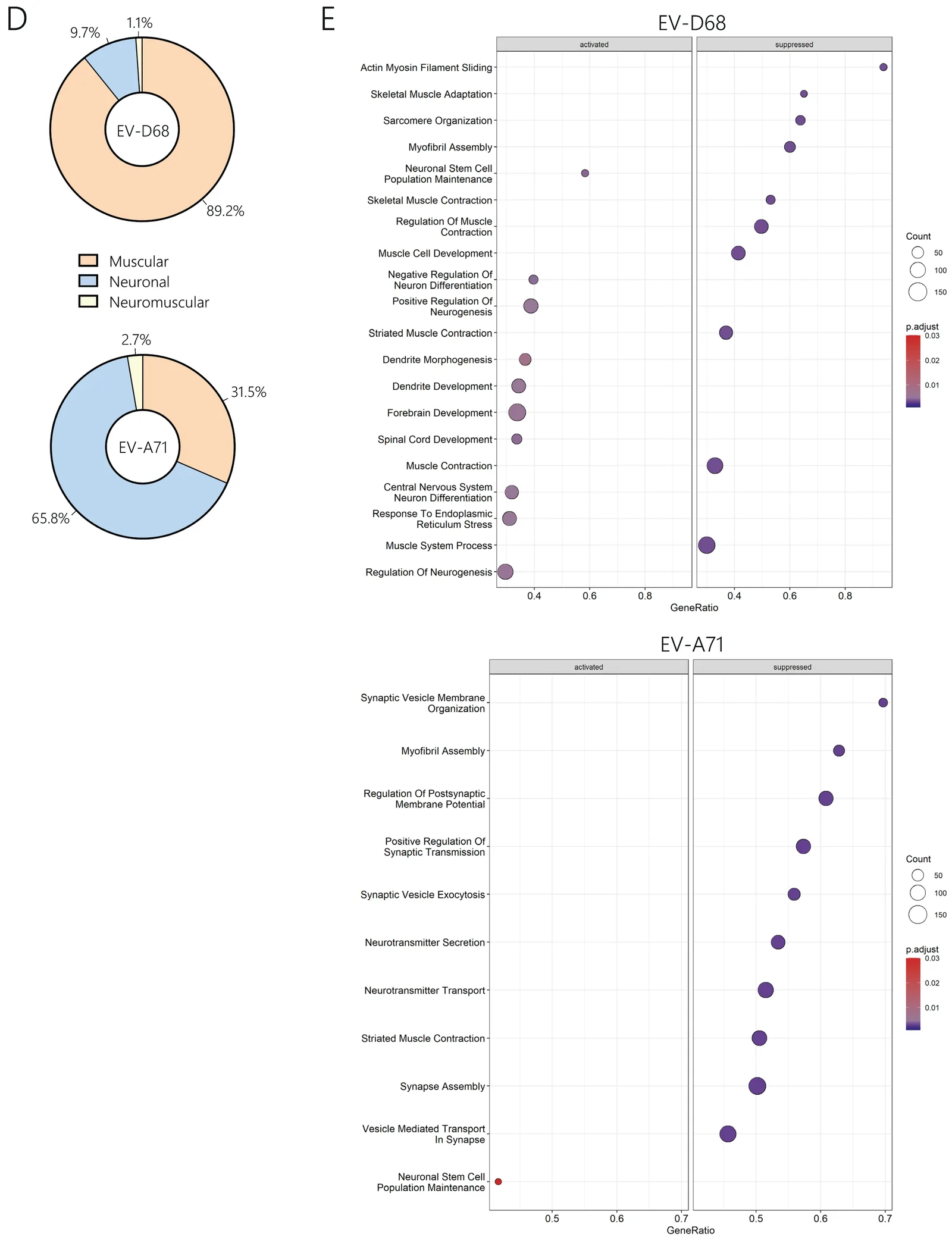
EV-A71 and EV-D68 infection downregulate neuronal and muscular pathways in NMOs. (**A**) PCA of transcriptomic profiles from mock, EV-A71-, and EV-D68-infected NMOs. (**B**) Heatmap of scaled gene expression profiles from IMR90-4- and M001-derived NMOs under mock and infected conditions. (**C**) Volcano plots showing log_2_ fold change versus –log_10_ adjusted *p* values for EV-A71- and EV-D68-infected NMOs compared to mock controls. Significantly upregulated and downregulated genes (adjusted *p* < 0.05, Benjamini–Hochberg correction) are shown in blue and pink, respectively. Each dot represents an individual gene. (**D**) Distribution of significantly suppressed neuronal and muscular Gene Ontology (GO) biological processes identified by gene set enrichment analysis (GSEA). Pie charts indicate the relative proportion of downregulated pathways in infected NMOs compared to mock controls. (**E**) GSEA dot plots showing the main altered neuronal and muscular pathways following EV-A71 or EV-D68 infection.

After confirming that NMOs are permissive to EV-A71 and EV-D68 infection, we performed bulk transcriptomic profiling to assess infection-induced changes in gene expression. NMOs from two donors (IMR90-4 and M001) were infected and, after 96 hpi, used for bulk RNA sequencing. Principal component analysis (PCA; Figure 2A) showed that viral infection was the dominant source of transcriptomic variation after 96 hpi, accounting for over 60% of the variance, whereas cell line origin contributed comparatively little (PC2: 10–15%; Supplementary Figure S2A). Heatmap analysis of scaled gene expression values revealed pronounced transcriptional changes in EV-A71-infected samples relative to mock controls, whereas EV-D68-infected samples clustered more closely with mock controls, indicating a limited alteration consistent with lower infectivity. These trends were observed for both IMR90-4 and M001 cell lines (Figure 2B). Subsequent analysis was performed on pooled data from both hiPSC lines.

To assess the extent of transcriptional changes induced by EV-A71 and EV-D68 infection, we analysed differentially expressed genes (DEGs) in NMOs. We observed that EV-A71 infection resulted in a substantially broader transcriptional response, with 1819 DEGs (1019 downregulated and 700 upregulated), compared to only 267 DEGs following EV-D68 infection (250 downregulated and 17 upregulated) (Figure 2C). This difference in DEG burden was consistent across both hiPSC lines, as visualised by Venn diagrams (Supplementary Figure S2B) showing shared and unique DEGs for each virus, with overall expression patterns illustrated in Supplementary Figure S2C.

To move beyond individual gene changes and understand the functional consequences of infection, we performed gene set enrichment analysis (GSEA). Using the full GO Biological Process library, both viruses activated pathways related to (m)RNA processing, as expected during viral infection (Supplementary Figure S2D). When specifically exploring neuromuscular-relevant gene sets, GSEA revealed suppression of both neuronal and muscular pathways following infection. Notably, EV-A71 predominantly suppressed neuronal pathways, whereas EV-D68 exerted a stronger effect on muscle-associated pathways (Figure 2D). To assess overlap in suppressed pathways, we compared the number of shared and unique neuronal and muscular pathways affected by each virus. Most suppressed muscle-related pathways were shared between EV-A71 and EV-D68 (114 shared pathways, 13 pathways uniquely affected by EV-A71, and 26 pathways uniquely affected by EV-D68), while suppression of neuronal pathways was predominantly observed in EV-A71 infection, with limited overlap with EV-D68 (18 shared pathways and 137 pathways uniquely affected by EV-A71; Supplementary Figure S2E). The top 10 suppressed pathways further illustrate that EV-A71 predominantly downregulates neuronal pathways, particularly those involved in synaptic vesicle release and signalling (e.g., synaptic vesicle exocytosis, neurotransmitter secretion, and synaptic vesicle membrane organisation). EV-D68 mainly suppressed muscle-related pathways, specifically those associated with skeletal (striated) muscle structure and function (e.g., sarcomere organisation, actin myosin filament sliding, and muscle contraction). Interestingly, neuronal pathways associated with development and differentiation were activated upon EV-D68 infection (Figure 2E).

In summary, EV-A71 and EV-D68 infection in NMOs induced distinct transcriptomic alterations, leading to the suppression of key neuronal and muscular pathways. These findings provide insight into how these viruses may contribute to neuromuscular dysfunction during infection.

### 3.3. EV-A71 and EV-D68 Stochastically Cleave SNAP25

Given the observed downregulation of synaptic pathways in infected NMOs in the bulk RNA-seq analysis, we next investigated whether viral infection affected synaptic vesicle release at the NMJ. Neurotransmitter release at the NMJ depends on the SNARE complex, composed of synaptobrevin, syntaxin-1, and synaptosomal-associated protein 25 (SNAP25), which mediates vesicle fusion and acetylcholine (ACh) release into the synaptic cleft [29,30,31]. Although EVs have been reported to interact with other SNARE proteins, such as cleavage of SNAP29 by EV-D68 and Coxsackievirus B3 to evade intracellular degradation [45,46], and SNAP23 being essential in EV-D68 replication organelle formation [47], SNAP25 is the principal presynaptic SNARE at the NMJ and a direct determinant of neurotransmitter release. Therefore, based on the RNA-seq-identified suppression of synaptic pathways, we assessed whether EV-A71 and EV-D68 infection affected SNAP25 integrity.

**Figure 3:**
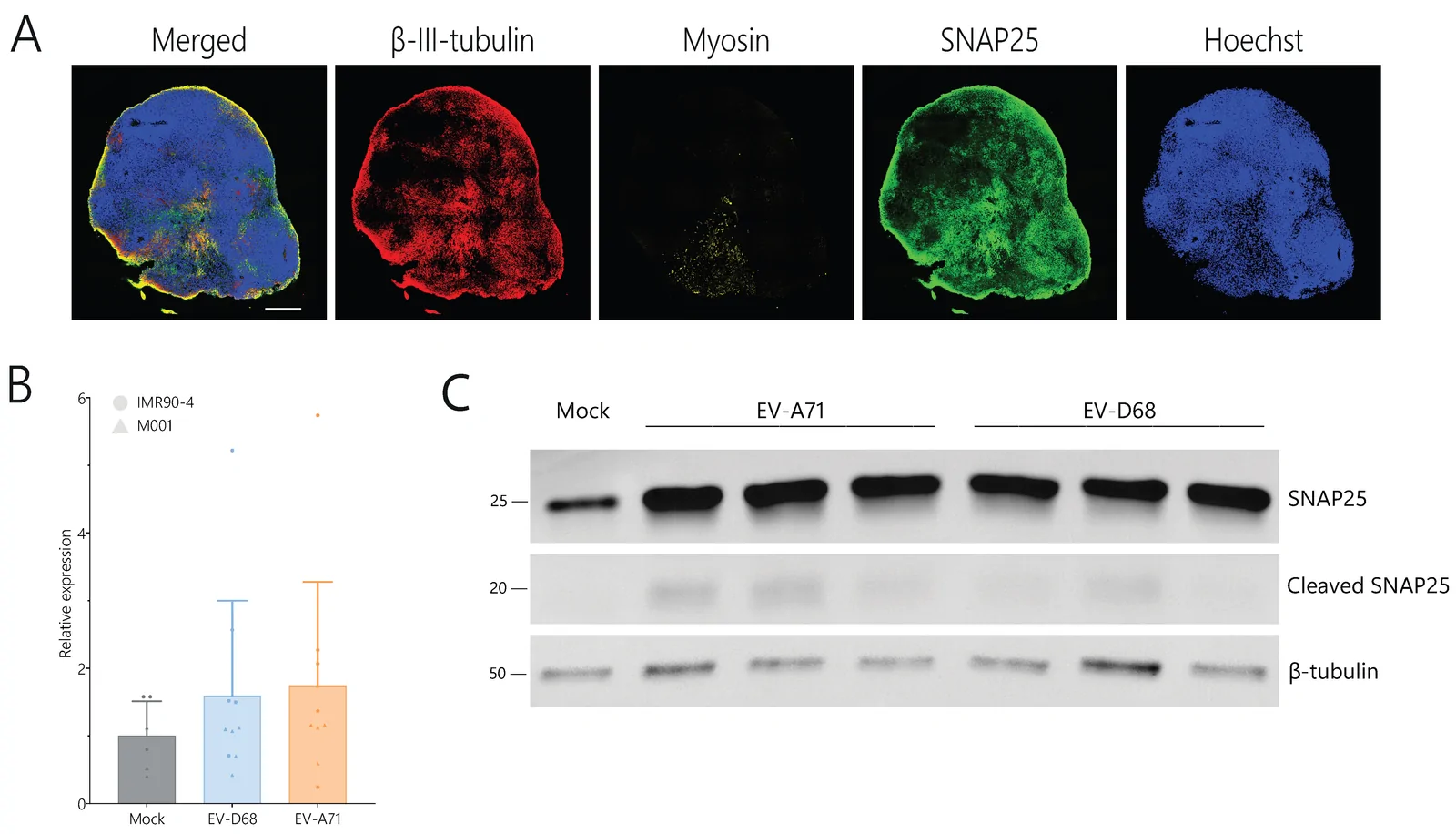
SNAP25 expression and cleavage in NMOs upon EV infection. (**A**) Representative immunofluorescence image of an IMR90-4-derived NMO stained for Hoechst, SNAP25, Fast MyHC, and β-III-tubulin (TUJ1). Scale bars: 300 µm. (**B**) Densitometric analysis of SNAP25 protein abundance in NMOs derived from two hiPSC lines (IMR90-4, circles; M001, triangles) following EV-A71 (*p* = 0.47) or EV-D68 (*p* = 0.99) infection. SNAP25 band intensities were quantified from Western blots and normalised to the loading control and mock condition (set to 1). Data represent the geometric mean of three technical replicates from two independent experiments. Statistical significance was assessed per virus using a Kruskal–Wallis test with Dunn’s multiple comparisons test. (**C**) Representative Western blot showing SNAP25 cleavage in M001-derived NMOs at 96 hpi following infection with EV-A71 or EV-D68.

The presence of SNAP25 in neurons was confirmed by co-localisation with β-III-tubulin (TUJ1), a neuronal marker, in NMOs (Figure 3A and Supplementary Figure S3A). To determine whether EV-A71 and EV-D68 infection affects SNAP25, NMOs were infected with each virus, and SNAP25 protein levels and cleavage fragments were assessed by Western blot. SNAP25 appeared more abundant in infected samples, but quantitative analysis revealed no statistically significant differences between infected and mock NMOs (Figure 3B). Cleaved SNAP25 fragments were detected at 96 hpi following infection with both EV-A71 and EV-D68 (Figure 3C), with a greater number of EV-A71-infected samples exhibiting detectable cleavage (EV-D68: 0/2 samples at 72 hpi, 2/12 at 96 hpi, 0/2 at 168 hpi; EV-A71: 0/2 at 72 hpi, 3/12 at 96 hpi, 1/2 at 168 hpi; Supplementary Figure S3B for 168 hpi data).

To validate the detection of cleaved SNAP25, NMOs and characterised hiPSC-MNs (Supplementary Figure S3C) were treated with botulinum neurotoxin A (BoNT-A, commonly known as Botox), a known SNAP25-cleaving protease [48]. BoNT-A treatment resulted in robust SNAP25 cleavage in both systems (Supplementary Figure S3D), confirming antibody specificity.

These findings demonstrate that SNAP25 is expressed in the neuronal compartment of NMOs, and both EV-A71 and EV-D68 infection can induce detectable, albeit infrequent, SNAP25 cleavage in NMOs.

### 3.4. EV-A71 and EV-D68 Infection Increases Cleaved Caspase-3

Transcriptomic analysis revealed enrichment of apoptosis-related pathways in both EV-A71- and EV-D68-infected NMOs, prompting further investigation of cell death signalling. Neuronal loss has been implicated in EV-associated paralytic disease [49,50], although EVs are known to modulate apoptotic pathways at different stages of infection, initially suppressing cell death to enhance viral replication and subsequently inducing apoptosis to facilitate viral release [51,52,53]. To determine whether EV-A71 and EV-D68 have an effect on cell death signalling in NMOs, we analysed the presence of cleaved caspase-3 (CC3).

**Figure 4:**
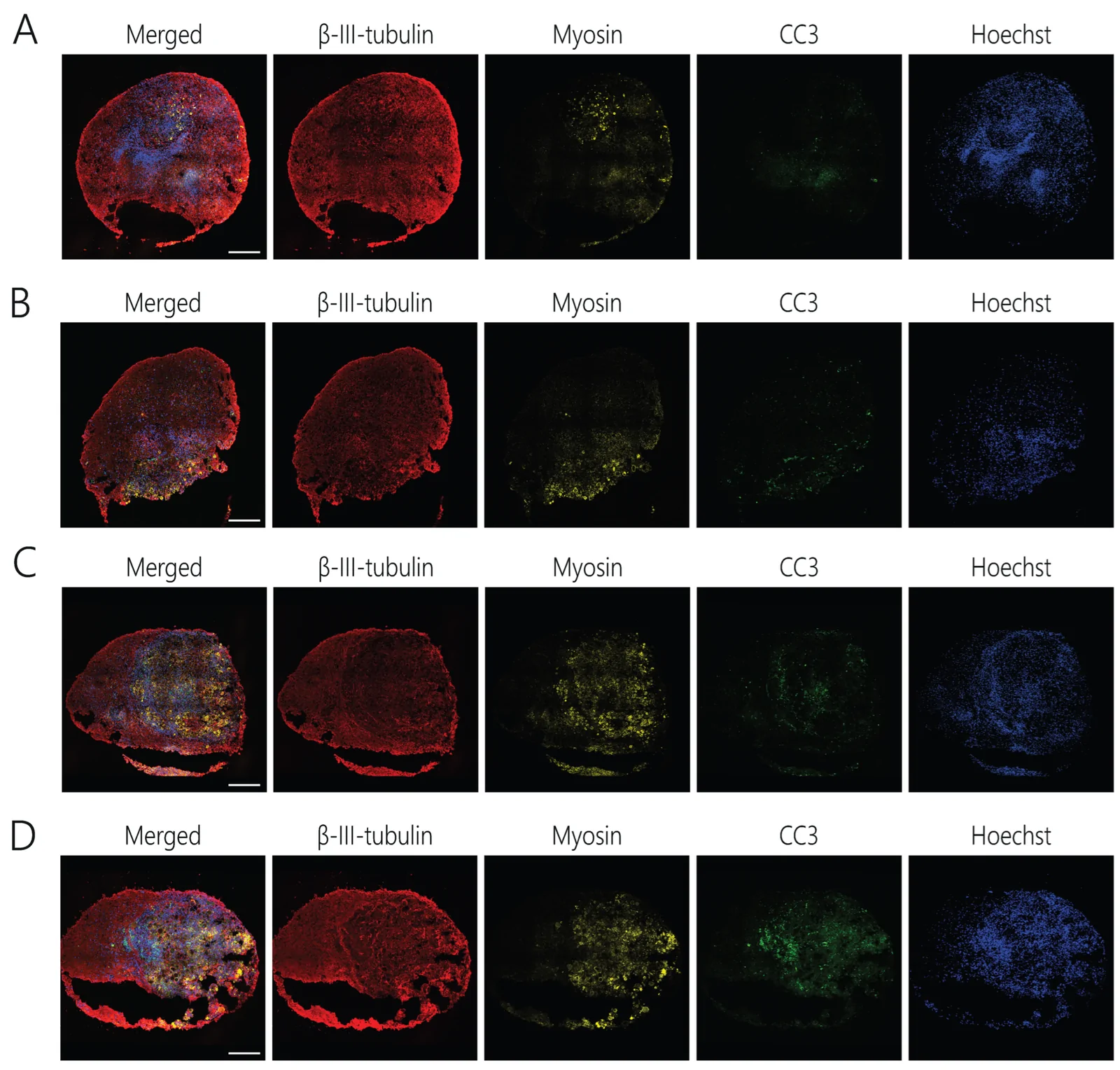
Caspase-3 activation in NMOs following staurosporine treatment or EV infection. (**A**) Representative immunofluorescence image of an IMR90-4-derived NMO stained for Hoechst, cleaved CC3, green, Fast MyHC, and β-III-tubulin (TUJ1); (**B**–**D**) NMOs treated with staurosporine (**B**) or infected with EV-A71 (**C**) or EV-D68 (**D**) for 72 h and stained for Hoechst, cleaved CC3, green, Fast MyHC, and β-III-tubulin (TUJ1). Scale bars: 200 µm.

To assess caspase-3 activation in our system, we performed CC3 immunostaining in infected NMOs. CC3-positive cells were detected in mock-treated NMOs (Figure 4A), predominantly in central regions, likely reflecting hypoxia-associated cell death frequently observed in neural organoids [54]. Treatment with staurosporine, a known apoptosis-inducing agent [55,56], induced a modest increase in CC3-positive cells, consistent with activation of apoptotic signalling (Figure 4B). Following infection with EV-A71 or EV-D68 for 72 h, NMOs exhibited a modest increased CC3 immunoreactivity compared to mock controls (Figure 4C,D), suggesting activation of caspase-3-associated signalling pathways.

These data demonstrate that EV-A71 and EV-D68 infection is associated with increased caspase-3 cleavage in NMOs.

## 4. Discussion

Non-polio EVs, such as EV-A71 and EV-D68, are increasingly recognised as causative agents of severe neurological complications, notably AFM. Despite their clinical significance, the molecular mechanisms underlying their neurovirulence remain incompletely understood. In this study, we investigated the effects of EV-A71 and EV-D68 infection on neuromuscular integrity using NMOs, which recapitulate key aspects of NMJ development and function.

Our results establish NMOs as a physiologically relevant in vitro model that is susceptible to EV-A71 and EV-D68 infection, particularly within neuronal populations. Previous studies have demonstrated that both viruses efficiently infect hiPSC-derived neurons in co-culture with astrocytes [25]. For EV-A71, clinical imaging and post-mortem analyses primarily indicate pathology centred on the spinal cord ventral horns and brainstem, consistent with MN involvement [57,58]. Experimental systems further demonstrate infection of mouse neuronal progenitor cells, with limited replication in differentiated neuronal lineages [59]. EV-A71 infection of human neuroblastoma cells and animal models (mouse and macaque) supports its neurotropic potential and association with paralytic disease [57,60]. EV-D68 has similarly been shown to infect human and mouse neuroblastoma cells, hiPSC-derived cortical neurons, and primary murine astrocytes [61,62], as well as human cerebral organoids [63]. More recently, EV-D68 infection has been demonstrated in MNs, neurons, astrocytes, and oligodendrocyte progenitors within spinal cord organoids [64,65], highlighting its capacity to target multiple neural lineages. These findings indicate that, in terms of neuronal populations, EV-A71 and EV-D68 share broadly overlapping tropism, with both viruses being able to infect neurons and support glial cells.

Transcriptomic analysis of NMOs revealed downregulation of neuronal and muscular gene networks following infection with either virus. EV-A71 infection resulted in suppression of a greater number of neuronal pathways compared to EV-D68, whereas both viruses exerted broadly comparable effects on muscle-associated pathways. These findings suggest a distinct impact on neuromuscular function. It should be noted that EV-D68 replication was less efficient compared to EV-A71 replication in NMOs. The EV-D68 experiments were performed using the prototype Fermon strain, originally isolated in 1962 and widely used in the EV-D68 research field. In contrast, the EV-A71 strain used in this study belongs to the C2 genotype, which has been associated with neurological disease, including cases of AFM [66]. The use of the historic Fermon strain, rather than contemporary neurotropic EV-D68 isolates associated with AFM outbreaks, is a limitation of this study. Therefore, differences in replication kinetics and host responses observed between EV-A71 and EV-D68 in our model may in part reflect strain-specific characteristics. Future studies using contemporary EV-D68 outbreak isolates will be necessary to determine whether similar effects are observed in clinically relevant neurotropic EV-D68 strains. Nonetheless, the observed global transcriptional changes are consistent with prior reports from mouse and human studies indicating that both EV-A71 and EV-D68 exhibit neurotropism and myotropism. Clinically, EV-A71 disease is dominated by neurotropic manifestations, including brainstem encephalitis and polio-like paralysis, as documented in human cases [12,13,14,21,58] and recapitulated in mouse models [17,18,19,20]. In contrast, clear muscle-targeted pathology for EV-A71 has only been reported in experimental mouse studies [67,68]. EV-D68, by comparison, has been strongly associated with outbreaks of AFM, in which pathology is usually clinically centred on anterior horn cells of the spinal cord and loss of MNs [15]. EV-D68 infection of human iPSC-derived MNs has also been demonstrated in vitro [22]. While clinical and pathological series in humans continue to emphasize spinal MN injury, evidence from mouse models [23,24] and more recent studies using human iPSC-derived skeletal muscle systems [25] indicate pronounced EV-D68 myotropism and muscle fibre damage.

As there was a decrease in genes related to the synaptic pathways in EV-infected NMOs, we investigated the role of SNAP25, a critical SNARE protein involved in synaptic vesicle fusion. Infection with EV-A71 or EV-D68 was associated with sporadic SNAP25 cleavage, with detectable fragments more frequently observed in EV-A71-infected NMOs. Previous studies have demonstrated cleavage of SNAP29, a SNARE protein involved in autophagy, by EV-D68 in cell lines [45,46], and a role for SNAP23 in EV-D68 replication organelle formation [47]. Our findings extend these observations by suggesting that enteroviruses may also target SNAP25 in a neuromuscular context, pointing to a potential mechanism of synaptic disruption that has not previously been described. We also observed a modest increase in CC3 immunoreactivity in NMOs following infection with both EV-A71 and EV-D68, suggesting activation of caspase-3-associated cell death pathways. Prior studies in cell culture models have reported induction of apoptotic signalling during EV-A71 and EV-D68 infection, including activation of caspase-3 and pro-apoptotic factors such as Bax and Bak [51,52,53,69,70]. However, studies on poliovirus (PV) has demonstrated that caspase activation during infection can also result from direct cleavage by viral proteases, including 2A and 3C, thereby complicating the interpretation of canonical apoptosis markers [71,72,73]. Thus, while increased CC3 staining in our model is consistent with engagement of apoptotic signalling, further investigation is required to determine whether this reflects bona fide apoptosis or virus-modulated cell death mechanisms. Activation of apoptotic signalling together with sporadic SNAP25 cleavage may act synergistically to exacerbate neuronal dysfunction and tissue damage, potentially contributing to paralysis in only a subset of infected individuals. This would add a potential molecular bottleneck contributing to the rare induction of paralytic disease, paralleling the highly dynamic and stochastic process previously proposed for poliovirus trafficking from the gut to the CNS [74].

It is important to highlight that batch-dependent variability of NMOs necessitates cautious interpretation of these results. For instance, SNAP25 cleavage upon EV-A71 infection was more frequently observed in M001-derived NMOs, and differences in gene expression pathways were noted between M001 and IMR90-4-derived NMOs. Additionally, as with all in vitro models, NMOs do not fully recapitulate the complexity of in vivo neuromuscular systems, and functional correlates of the observed molecular changes remain to be established. Nevertheless, the use of an isogenic model containing distinct neuronal and muscular regions to study EV infection is an essential advance in our understanding of these pathogens. The use of such a multilineage system is warranted based on clinical observations of post-polio syndrome, where a pattern of muscle involvement is thought to occur secondary to neuronal dysfunction. For example, Bickerstaffe et al. [75] demonstrated significant muscle loss in post-polio syndrome patients using quantitative muscle ultrasound and strength measurements. Drawing such parallels and using complex multilineage systems will help to better understand the complex interplay between neuronal infection and muscle pathology in enteroviral diseases.

Overall, our findings provide valuable insights into the pathogenic molecular mechanisms underlying AFM following EV infection. Future studies should aim to resolve the cell-type-specific and spatial dynamics of infection within NMOs. In particular, single-cell RNA sequencing and spatial transcriptomics would enable identification of the specific neuronal and muscular subpopulations that are infected and reveal how viral infection reshapes transcriptional programs at cellular resolution. In addition, longitudinal analyses are required to define the temporal dynamics of viral spread and to determine how infection evolves within distinct cell populations over time. This is particularly relevant for understanding the dynamics of SNAP25 protein cleavage and caspase-3-associated apoptotic signalling, which in the present study were observed as sporadic and spatially heterogeneous events. Functional studies, including electrophysiological measurements and quantitative assessment of contractile activity, will be important to link these molecular changes to neuromuscular output. Finally, mechanistic studies to identify the viral proteases and/or host factors responsible for SNAP protein cleavage, and the exploration of SNAP25 cleavage as a potential biomarker of synaptic dysfunction may provide additional insight into virus-induced neuromuscular impairment, although its potential as therapeutic target remains to be established.

## Figures and Tables

**Table 1:** List of the primary and secondary antibodies used for the immunofluorescence staining.

**Target Protein**	**Species Origin**	**Company**	Cat. # *	**Dilution**	**Specificity**
β-III-tubulin (TUJ1)	Chicken	Abcam, Cambridge, UK	AB41489	1:500	Neuronal cytoskeleton/neuronal identity marker
	Rabbit	Invitrogen, Waltham, MA, USA	PA5-86069	1:200	
Choline acetyltransferase	Goat	Sigma-Aldrich, Saint Louis, MO, USA	AB144	1:200	Cholinergic motor neuron marker (ACh synthesis enzyme)
Fast MyHC/Myosin	Mouse	Sigma-Aldrich, Saint Louis, MO, USA	M1570	1:200	Muscle structural protein
	Rabbit	Sigma-Aldrich, Saint Louis, MO, USA	M7523	1:200	
dsRNA (clone rJ2)	Mouse	Sigma-Aldrich, Saint Louis, MO, USA	MABE1134	1:60	Viral replication intermediate (active RNA virus replication)
SNAP25	Rabbit	Sigma-Aldrich, Saint Louis, MO, USA	S9684	1:5000	Presynaptic SNARE protein (synaptic vesicle fusion)
CC3	Rabbit	Cell Signaling Tech., Danvers, MA, USA	9661	1:200	Apoptosis marker (cleaved caspase-3 activation)
**Secondary Antibody**	**Species origin**	**Company**	Cat. # *	**Dilution**	
Anti-Chicken ALEXA 647	Donkey	ThermoFisher Scientific, Waltham, MA, USA	A78952	1:1000	
Anti-Mouse ALEXA 546	Donkey	ThermoFisher Scientific, Waltham, MA, USA	A10036	1:1000	
Anti-Rabbit ALEXA 488	Donkey	ThermoFisher Scientific, Waltham, MA, USA	A21206	1:1000	
Anti-Goat ALEXA 680	Donkey	ThermoFisher Scientific, Waltham, MA, USA	A21084	1:1000	
Anti-α-BTX + Alexa Fluor 647		Invitrogen, Waltham, MA, USA	B35450	1:1000	

* Cat. # stands for Catalogue Number.

**Table 2:** List of the primary and secondary antibodies used for the Western blot.

Target Protein	Species Origin	Company	Cat. # *	Dilution
β-tubulin	Mouse	Invitrogen, Waltham, MA, USA	MAS16308	1:2000
SNAP25 N-terminal	Rabbit	Sigma-Aldrich, Saint Louis, MO, USA	S9684	1:5000
	Mouse	BioLegend, San Diego, CA, USA	836304	1:2000
HSP60	Rabbit	Abcam, Cambridge, UK	AB46798	1:2000
Anti-Mouse HRP	Goat	Bio-Rad Laboratories, Hercules, CA, USA	1706516	1:2000
Anti-Rabbit HRP	Goat	Bio-Rad Laboratories, Hercules, CA, USA	1706515	1:2000

* Cat. # stands for Catalogue Number.

**Table 3:** Sequences (5′–3′) of the primers and probes used in this study.

Target Gene	Primer Type	Sequence	Genome Position	Probe Labels
EV-D68 (AY426531)	Forward	TGT TCC CAC GGT TGA AAA CAA	nt 204–220 (+ strand)	None
	Reverse	TGT CTA GCG TCT CAT GGT TTT CAC	nt 382–405 (− strand)	None
	Probe 1	ACC GCT ATA GTA CTT CG	nt 232–248 (+ strand)	5′ 6FAM–3′MGB NFQ
	Probe 2	TCC GCT ATA GTA CTT CG	nt 233–248 (+ strand)	5′ 6FAM–3′MGB NFQ
EV-A71 (FJ172159)	Forward	GGC CCT GAA TGC GGC TAA T	nt 417–433 (+ strand)	
	Reverse	GGG ATT GTC ACC ATA AGC C	nt 548–563 (− strand)	

## Data Availability

The raw data supporting the conclusions of this article will be made available by the authors on request.

## Supplementary Materials

The following supporting information can be downloaded at https://www.mdpi.com/article/10.3390/v18080853/s1, Figure S1: Immunofluorescence characterization of NMOs from different hiPSC lines; Figure S2: Additional transcriptomic analyses of EV-A71 and EV-D68 infected NMOs; Figure S3: EV-A71 and EV-D68 induce sporadic cleavage of SNAP25 in NMOs; Video S1: NMO spontaneously contracting.

## Abbreviations

The following abbreviations are used in this manuscript:

AFM	acute flaccid myelitis
NMOs	neuromuscular organoids
NMJ	neuromuscular junction
MN	motor neuron
hiPSC	human induced pluripotent stem cell
hpi	hours post infection
DEGs	differentially expressed genes
GSEA	gene set enrichment analysis
GO	gene ontology
CC3	cleaved caspase-3
ACh	acetylcholine
dsRNA	double-stranded RNA
Fast MyHC	fast myosin heavy chain
PCA	principal component analysis
HFMD	hand, foot, and mouth disease
NMO	neuromuscular organoid
