# Supplementary material for: Non-Polio Enterovirus A71 and D68 Infection of Human Neuromuscular Organoids Reveals Distinct Mechanisms of Neuromuscular Impairment
Source: Viruses. 2026 Aug 4;18(8):853. doi: 10.3390/v18080853 (PMC13517452; doi:10.3390/v18080853)
Supplement: Supplementary file 1 [file viruses-18-00853-s001.zip › NMO-EV Supplementary Figures.pdf]

Article

Non-Polio Enterovirus A71 and D68 Infection of Human Neuromuscular Organoids Reveals Distinct Mechanisms of Neuromuscular Impairment

Amber J. Schotting [1,2,†], Inés García-Rodríguez [1,2,†], Eline Freeze [1,2], Anoop T. Ambikan [3], Michael Wagner [4], Mira Mioch [1,2], Aymeric P. Y. L. Moffelein [1,2], William Jackson [4], Dasja Pajkrt [1,5], Katja C. Wolthers [2], Renata Vieira de Sá [2,‡] and Adithya Sridhar [1,2,5,*,‡]

[1] OrganoVIR Labs, Department of Paediatric Infectious Diseases, Emma Children's Hospital, Amsterdam Institute for Reproduction and Development, Amsterdam Institute for Infection and Immunity, Amsterdam UMC, Location Academic Medical Center, University of Amsterdam, Meibergdreef 9, 1105 AZ Amsterdam, The Netherlands
[2] OrganoVIR Labs, Department of Medical Microbiology and Infection Prevention, Amsterdam Institute for Infection and Immunity, Amsterdam UMC, Location Academic Medical Center, University of Amsterdam, Meibergdreef 9, 1105 AZ Amsterdam, The Netherlands
[3] The Systems Virology Lab, Department of Laboratory Medicine, Division of Clinical Microbiology, Karolinska Institute, ANA Futura, Campus Flemingsberg, 17177 Stockholm, Sweden
[4] Department of Microbiology and Immunology and Center for Pathogen Research, University of Maryland School of Medicine, Baltimore, MD 21201, USA
[5] Emma Center for Personalized Medicine, Amsterdam UMC, Meibergdreef 9, 1105 AZ Amsterdam, The Netherlands
* Correspondence: a.sridhar@amsterdamumc.nl
† These authors contributed equally to this work.
‡ These authors also contributed equally to this work.

Supplementary Figures

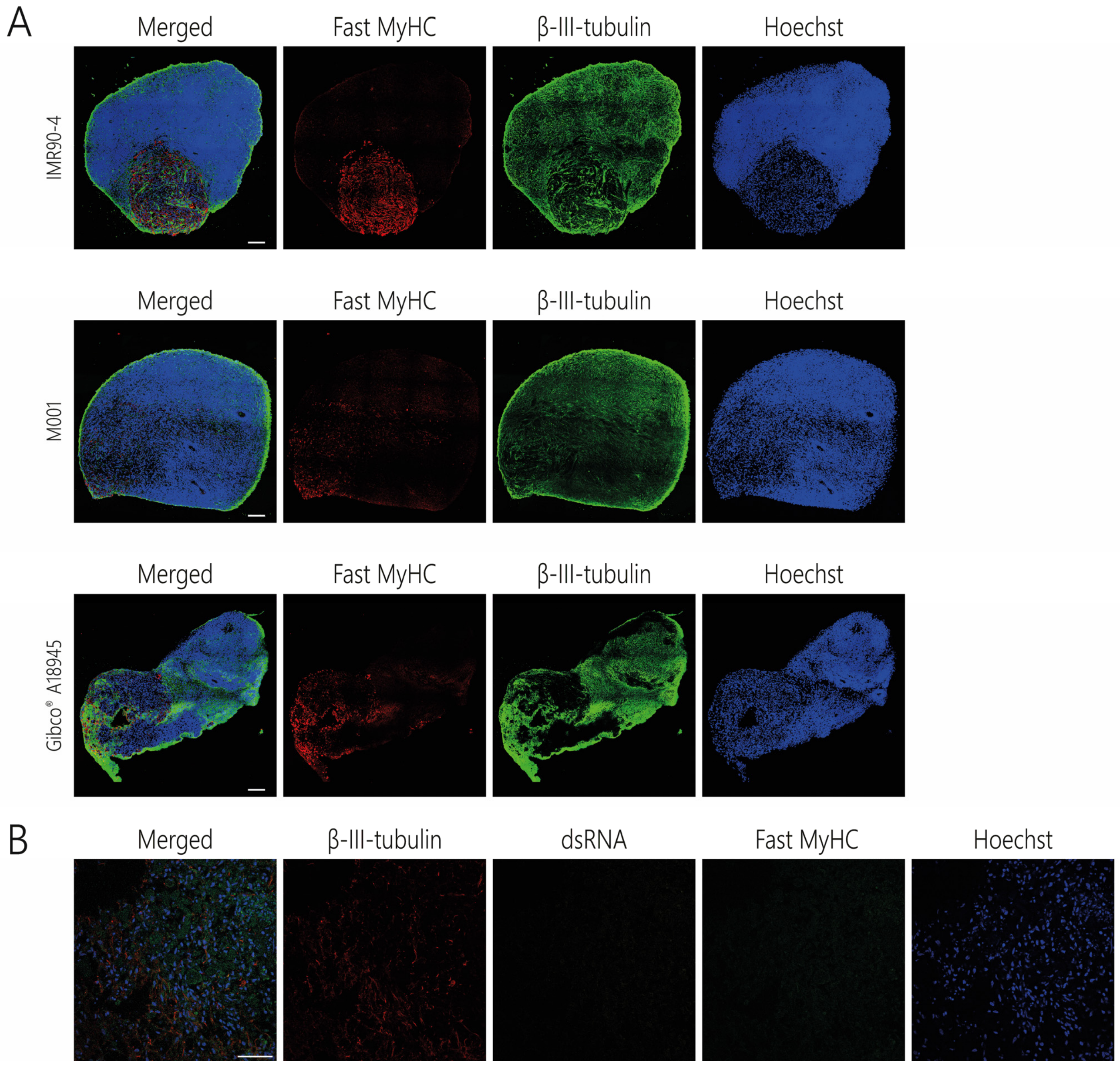

Supplementary Figure S1. Immunofluorescence characterization of NMOs from different hiPSC lines. **(A)** Immunofluorescence images of NMO derived from different hiPSC lines stained for Hoechst, β-III-tubulin and fast myosin heavy chain (Fast MyHC). Scale bars: 100 μm. **(B)** Immunofluorescence image of a mock infected Gibco® A18945-derived NMO stained for Hoechst, Fast MyHC, dsRNA, and β-III-tubulin. Scale bars: 50 μm.

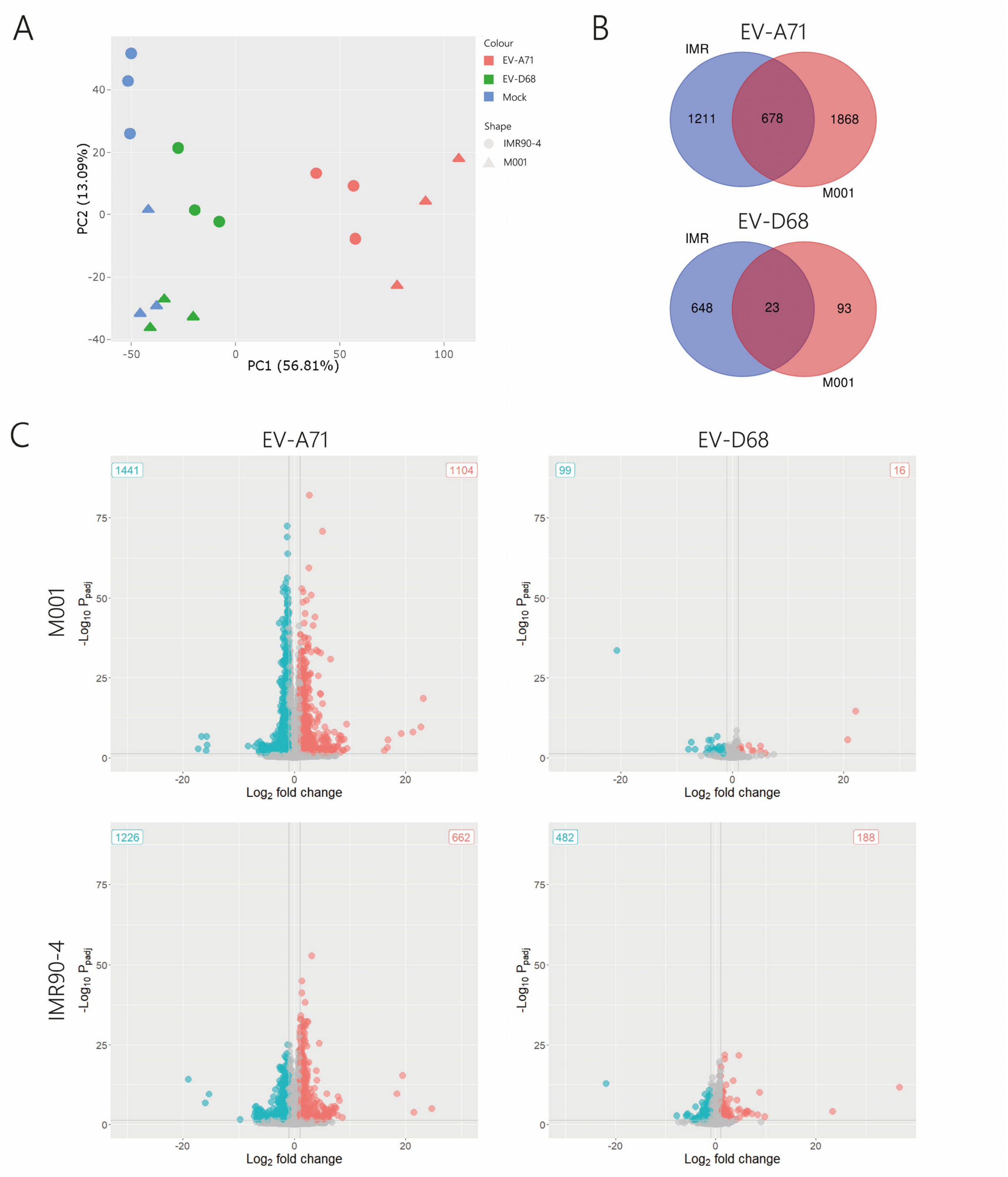
A
PC2 (13.09%)
PC1 (56.81%)
Colour
EV-A71
EV-D68
Mock
Shape
IMR90-4
M001
B
EV-A71
IMR
M001
1211
678
1868
EV-D68
IMR
M001
648
23
93
C
EV-A71
EV-D68
M001
IMR90-4
1441
1104
99
16
1226
662
482
188
-Log10 Ppadj
Log2 fold change

D

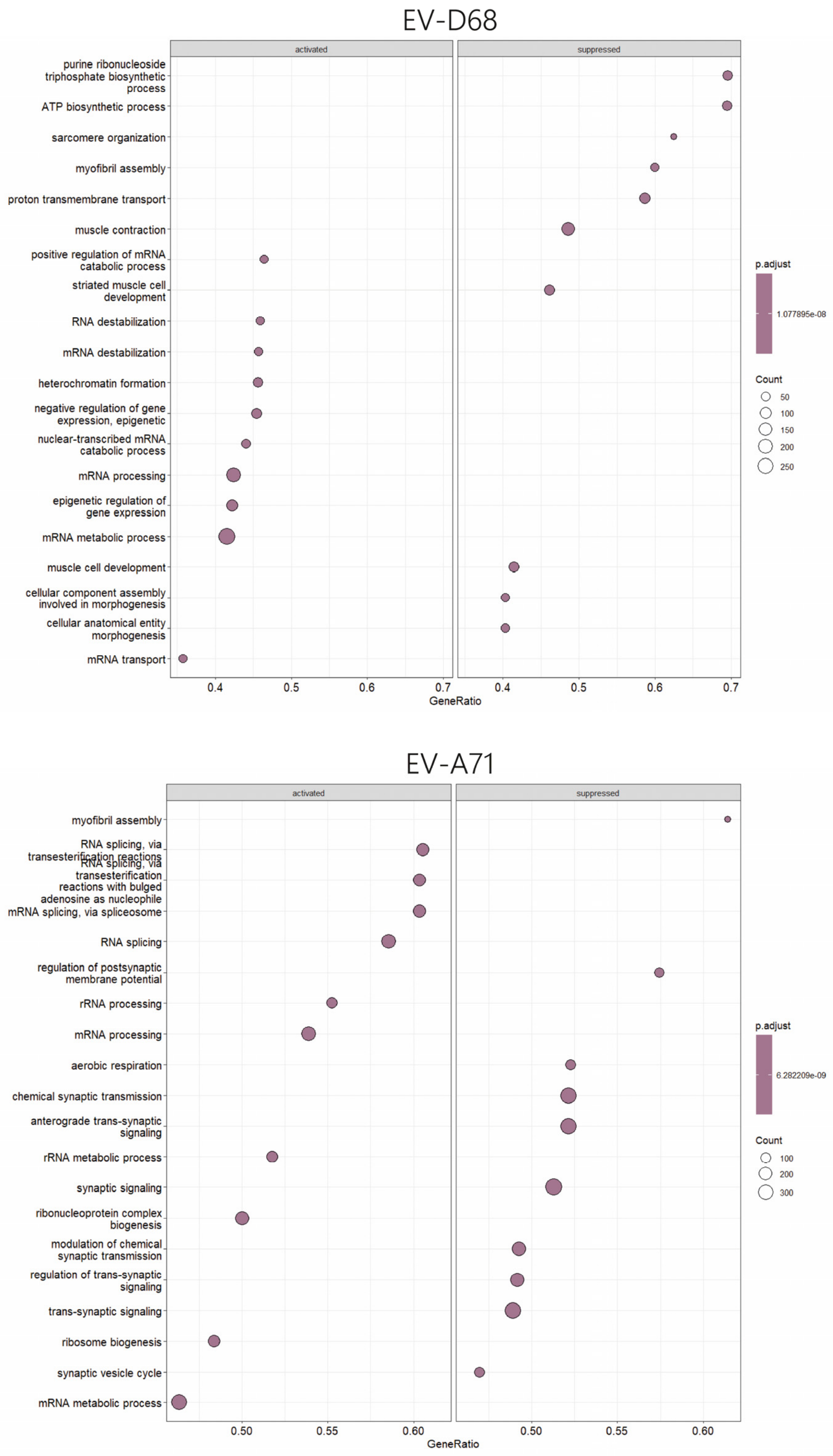
EV-D68
activated
suppressed
purine ribonucleoside triphosphate biosynthetic process
ATP biosynthetic process
sarcomere organization
myofibril assembly
proton transmembrane transport
muscle contraction
positive regulation of mRNA catabolic process
striated muscle cell development
RNA destabilization
mRNA destabilization
heterochromatin formation
negative regulation of gene expression, epigenetic
nuclear-transcribed mRNA catabolic process
mRNA processing
epigenetic regulation of gene expression
mRNA metabolic process
muscle cell development
cellular component assembly involved in morphogenesis
cellular anatomical entity morphogenesis
mRNA transport
0.4
0.5
0.6
0.7
GeneRatio
p.adjust
1.077895e-08
Count
50
100
150
200
250
EV-A71
myofibril assembly
RNA splicing, via transesterification reactions
RNA splicing, via transesterification reactions with bulged adenosine as nucleophile
mRNA splicing, via spliceosome
RNA splicing
regulation of postsynaptic membrane potential
rRNA processing
mRNA processing
aerobic respiration
chemical synaptic transmission
anterograde trans-synaptic signaling
rRNA metabolic process
synaptic signaling
ribonucleoprotein complex biogenesis
modulation of chemical synaptic transmission
regulation of trans-synaptic signaling
trans-synaptic signaling
ribosome biogenesis
synaptic vesicle cycle
mRNA metabolic process
0.50
0.55
0.60
GeneRatio
6.282209e-09
100
200
300

E

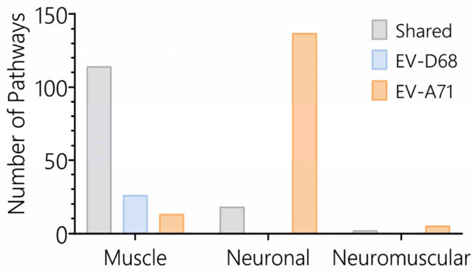
Number of Pathways
0
50
100
150
Shared
EV-D68
EV-A71
Muscle
Neuronal
Neuromuscular

Supplementary Figure S2. Additional transcriptomic analyses of EV-A71 and EV-D68 infected NMOs. **(A)** PCA of transcriptomic profiles from mock-, EV-A71–, and EV-D68–infected NMOs coloured by hiPSC line origin (IMR90-4 and M001). **(B)** Venn diagrams of DEGs identified in IMR90-4– and M001–derived NMOs following EV-A71 or EV-D68 infection compared with mock controls. **(C)** Volcano plots showing $\log_2$ fold change versus $-\log_{10}$ adjusted p values for IMR90-4– and M001–derived NMOs following EV-A71 or EV-D68 infection compared with mock controls. **(D)** GSEA dot plots of the full GO Biological Process library following EV-A71 or EV-D68 infection. **(E)** Bar graph showing the numbers of suppressed neuronal and muscle-related GO biological processes unique to or shared between EV-A71– and EV-D68–infected NMOs.

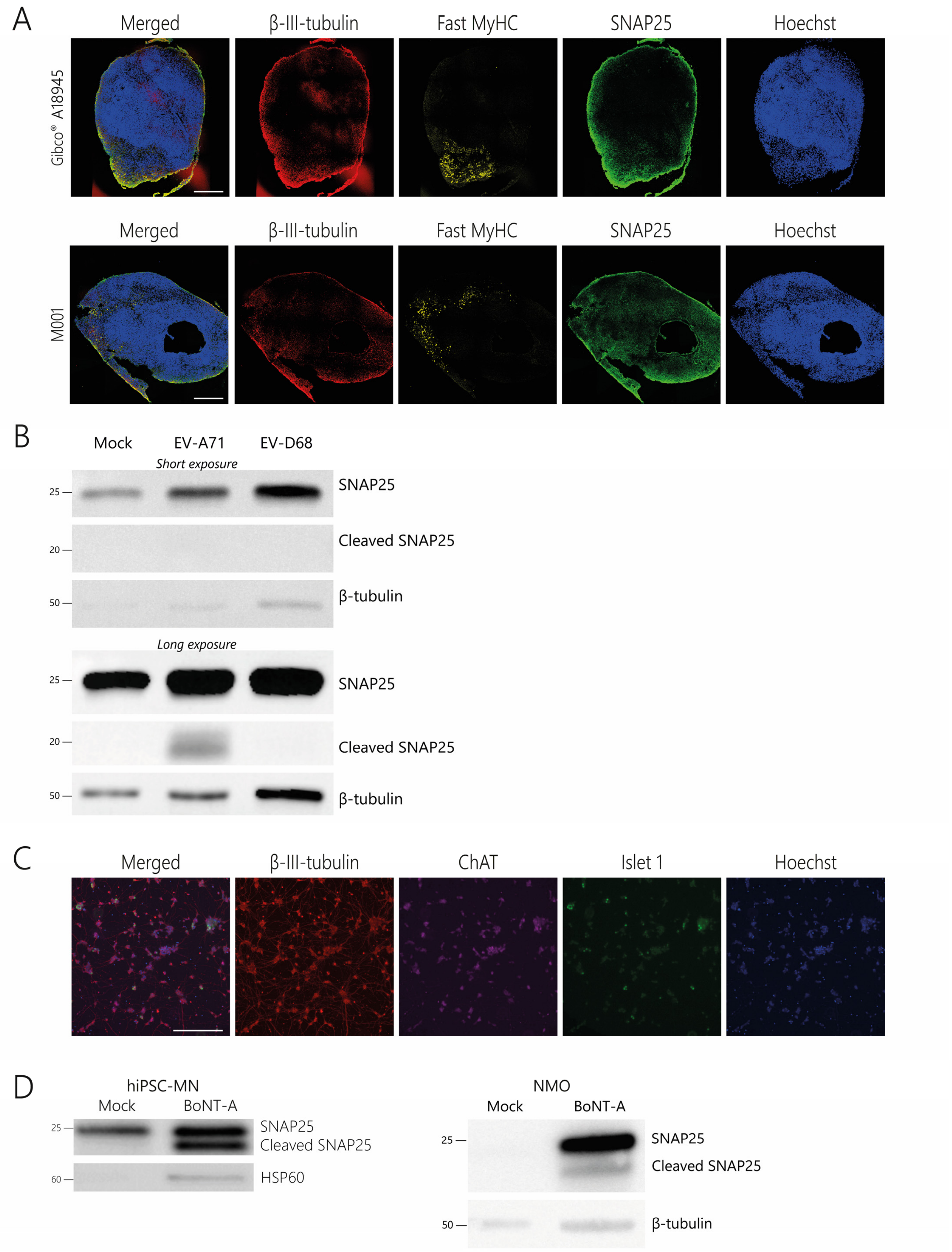

Supplementary Figure S3. EV-A71 and EV-D68 induce sporadic cleavage of SNAP25 in NMOs. **(A)** Immunofluorescence images of NMO derived from different hiPSC lines stained for Hoechst, SNAP25, β-III-tubulin and Fast MyHC. Scale bars: 200

μm. **(B)** Western blot detection of cleaved SNAP25 fragments in IMR90-4-derived NMOs at 168 hpi following infection with EV-A71 or EV-D68. **(C)** Immunofluorescence images of hiPSC-MN stained for Hoechst, ChAT, β-III-tubulin and Islet 1. Scale bars: 300 μm. **(D)** Validation of SNAP25 cleavage using BoNT-A treatment in hiPSC-MN and NMOs.

Supplementary Video's

Supplementary Video S1. NMO spontaneously contracting. Contraction of the muscle compartment in a neuromuscular organoid. Recording showing spontaneous and/or stimulated contractions of the muscle compartment within the neuromuscular organoid
